# The association between the CD4/CD8 ratio and surgical site infection risk among HIV-positive adults: insights from a China hospital

**DOI:** 10.3389/fimmu.2023.1135725

**Published:** 2023-07-11

**Authors:** Bo Liu, Kangpeng Li, Shengtao Li, Rugang Zhao, Qiang Zhang

**Affiliations:** Department of Orthopaedics, Beijing Ditan Hospital, Capital Medical University, Beijing, China

**Keywords:** association, CD4/CD8 ratio, surgical site infection, CD4 counts, HIV

## Abstract

**Purpose:**

It is well known that the CD4/CD8 ratio is a special immune-inflammation marker. We aimed to explore the relationship between the CD4/CD8 ratio and the risk of surgical site infections (SSI) among human immunodeficiency virus (HIV)-positive adults undergoing orthopedic surgery.

**Methods:**

We collected and analyzed data from 216 HIV-positive patients diagnosed with fractures at the department of orthopedics, Beijing Ditan Hospital between 2011 and 2019. The demographic, surgical, and hematological data for all patients were collected in this retrospective cohort study. We explored the risk factors for SSI using univariate and multivariate logistic regression analysis. Then, the clinical correlation between the CD4 count, CD4/CD8 ratio, and SSI was studied using multivariate logistic regression models after adjusting for potential confounders. Furthermore, the association between the CD4/CD8 ratio and SSI was evaluated on a continuous scale with restricted cubic spline (RCS) curves based on logistic regression models.

**Results:**

A total of 23 (10.65%) patients developed SSI during the perioperative period. Patients with hepatopathy (OR=6.10, 95%CI=1.46-28.9), HIV viral load (OR=8.68, 95%CI=1.42-70.2; OR=19.4, 95%CI=3.09-179), operation time (OR=7.84, 95%CI=1.35-77.9), and CD4 count (OR=0.05, 95%CI=0.01-0.23) were risk factors for SSI (P-value < 0.05). Our study demonstrated that a linear relationship between CD4 count and surgical site infection risk. In other words, patients with lower CD4 counts had a higher risk of developing SSI. Furthermore, the relationship between CD4/CD8 ratio and SSI risk was non-linear, inverse ‘S’ shaped. The risk of SSI increased substantially when the ratio was below 0.913; above 0.913, the risk of SSI was almost unchanged. And there is a ‘threshold-saturation’ effect between them.

**Conclusion:**

Our research shows the CD4/CD8 ratio could be a useful predictor and immune-inflammation marker of the risk of SSI in HIV-positive fracture patients. These results, from a Chinese hospital, support the beneficial role of immune reconstitution in HIV-positive patients prior to orthopedic surgery.

## Introduction

The acquired immunodeficiency syndrome (AIDS) epidemic, caused by the human immunodeficiency virus (HIV), is an ongoing global health crisis. HIV attacks and weakens the immune system, making it difficult for the body to fight infections and diseases (1, 2). According to the World Health Organization (WHO), as of 2021, there were an estimated 38 million people living with HIV worldwide, with the majority of cases occurring in sub-Saharan Africa (3). Despite significant progress in the development of effective antiretroviral therapies (ARTs) and prevention programs, HIV continues to be a major public health issue, especially in low- and middle-income countries. In 2021, the WHO estimated that there were approximately 2.3 million new HIV infections and 770,000 AIDS-related deaths worldwide. The WHO 2022–2030 global health sector strategy on HIV aims to reduce HIV infections from 1.5 million in 2020 to 335 000 by 2030, and deaths from 680 000 in 2020 to under 240 000 in 2030 (4).

HIV attacks the immune system and disrupts bone metabolism. Moreover, the side effects of oral antiviral drugs often lead to decreased bone density, and, the incidence of osteoporosis in AIDS patients is significantly increased (5). Patients with HIV often have various orthopedic diseases and require surgical treatment. However, because of the combination of immunodeficiency, malnutrition, multiple diseases (such as tuberculosis, syphilis, hepatitis C, and hepatitis B), and various opportunistic infections, many studies have shown that HIV-positive patients have a significantly higher risk of surgical site infections (SSI) than patients with normal orthopedic diseases (6–8).

The HIV/AIDS epidemic is characterized by a low prevalence rate, with an estimated 0.1% of the adult population living with HIV/AIDS; the number of infections is also increasing annually in China (9). Currently, in China the only effective treatment is antiretroviral therapy (ART), commonly known as “cocktail” therapy. This therapy also increases the number of CD4 T lymphocytes in patients with AIDS, rebuilds immune function, and reduces mortality. As part of a surgical treatment plan, patients should be fully evaluated before surgery, especially for CD4 T lymphocyte levels. Many studies have shown that CD4 T lymphocyte counts are negatively correlated with the risk of incision infection after orthopedic surgery (6, 10, 11). However, these studies had small sample sizes and many variables meaning that they were prone to large biases. In addition, data from Chinese hospitals is scarce, particularly for HIV-positive patients with fractures.

The CD4/CD8 ratio is a marker of immune dysfunction and systemic inflammation in the body and may be a good indicator of disease progression, treatment response, morbidity, and mortality (12). Further evidence suggests an overall increase in morbidity and mortality in HIV-positive individuals who fail to normalize their ratios (13, 14). Our study fills this gap by investigating the CD4/CD8 ratio as an independent predictor of surgical site infection risk among HIV-positive adults. By considering this specific immunological marker, we aim to provide novel insights into the underlying mechanisms of surgical site infections and potentially identify new avenues for intervention and prevention strategies. Furthermore, our study contributes to the existing literature by conducting the research within a Chinese hospital setting, which provides insights specific to this population. This geographical diversity adds value and expands the generalizability of the findings, as risk factors and outcomes may vary across different regions and healthcare settings.

As far as we know, little research has focused on the relationship between the CD4/CD8 ratio and the risk of SSI, and this issue has not yet been addressed. Compared with previous foreign studies, we used a larger sample size and recorded the variables for each patient in detail. Our goal was to explore the risk factors for incision site infection in orthopedic surgery by focusing on the relationship between the CD4/CD8 ratio and the risk of SSI. We hypothesized that a lower CD4/CD8 ratio in HIV-positive patients would increase the risk of SSI.

## Methods

### Data selection and study design

We collected and analyzed clinical data from the department of orthopedics, Beijing Ditan Hospital, affiliated Capital Medical University. As we all know, the Beijing Ditan Hospital is the National Medical Center for Infectious Diseases in China. This was a retrospective single-center cohort study. This study was approved by the Ethics Committee of the Beijing Ditan Hospital, Capital Medical University. All procedures involving human participants were performed in accordance with the institutional and/or National Research Council ethical standards and the 1964 Declaration of Helsinki and its subsequent amendments, or equivalent ethical standards.

**Figure 1:**
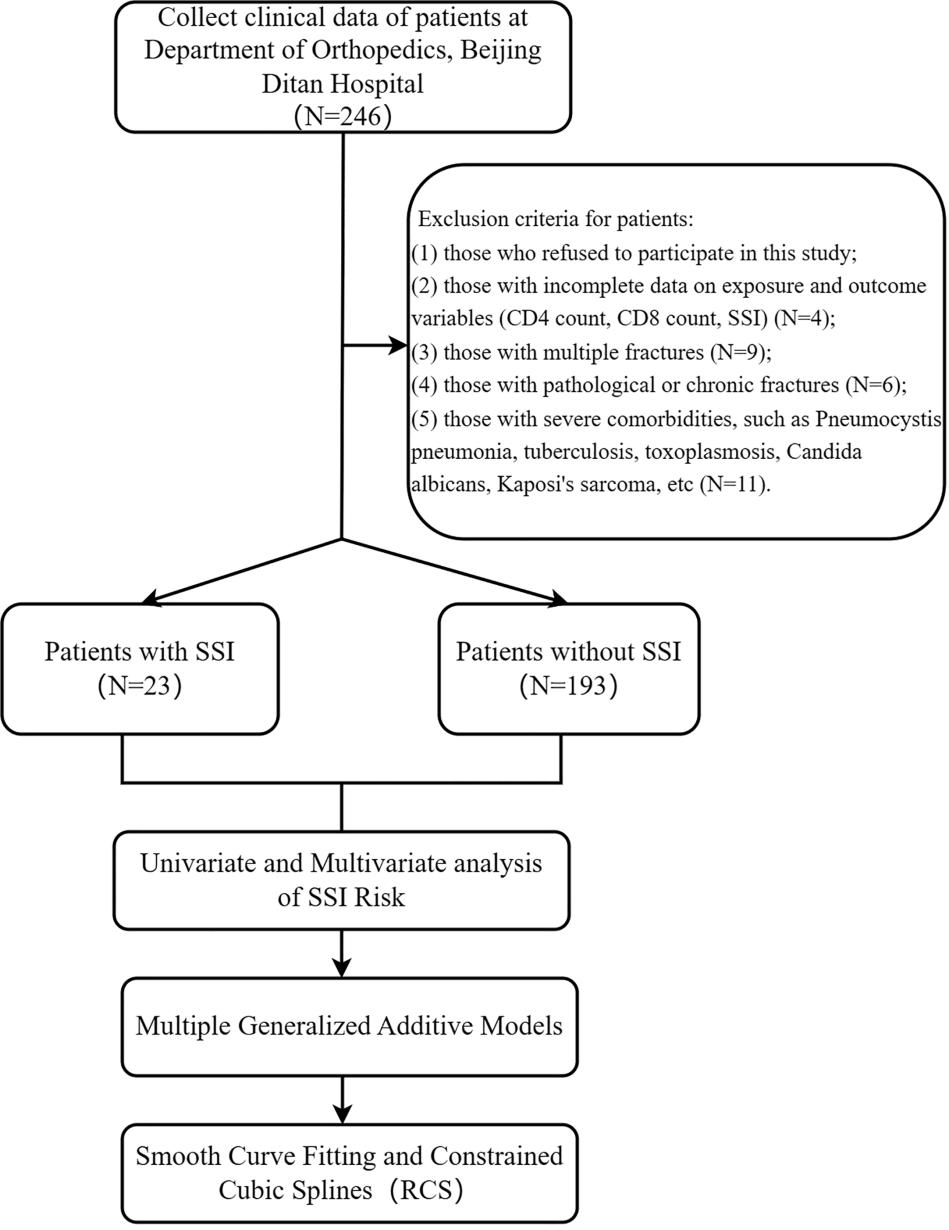
Flow chart for this study.

Inclusion criteria of patients in this study: (1) patients who were hospitalized in the Department of Orthopedics of Beijing Ditan Hospital between May 2011 and December 2019; (2) patients diagnosed as HIV-positive; (4) patients who received surgical treatment; (5)) Those receiving follow-up care within 3 to 12 months after surgery; (6) Those aged between 19 and 85. Exclusion criteria for patients: (1) those who refused to participate in this study; (2) those with incomplete data on exposure and outcome variables (CD4 count, CD8 count, SSI) (N=4); (3) those with multiple fractures (N=9); (4) those with pathological or chronic fractures (N=6); (5) those with severe comorbidities, such as Pneumocystis pneumonia, tuberculosis, toxoplasmosis, Candida albicans, Kaposi’s sarcoma, etc. (N=11). Ultimately, our cohort included 216 HIV-positive patients with fractures (**Figure 1**).

### Diagnostic criteria for HIV-positive patients

HIV-1/2 antibody testing is the gold standard for the diagnosis of HIV-infection, and the laboratory doctors use enzyme-linked immunoassay (ELISA) as a rapid method for detecting and diagnosing HIV patients in our hospital. They also use the 4th generation HIV kit (Abbott, UK) and the detection reagent Murex HIV Ag/Ab. For this study, we used a mini-VIDSA analyzer, Bio-Rad MODEL1575 plate washer, Axsym chemiluminescent immunoassay analyzer (Abbott, UK), and the ELECYS2010 chameleon enzyme immunoassay apparatus (Roche, Switzerland).

### Blood sample collection and processing

Fasting blood samples were collected by venipuncture using Vacutainer tubes containing EDTA (Becton Dickinson, Franklin Lakes, NJ, USA) for flow cytometry and morphological analysis. Before centrifugation, the serum samples were allowed to coagulate for 45 min. Then they were centrifuged at 3000 × *g* for 10 min. Serum aliquots were stored at -80°C.

### HIV viral load in plasma and cell count

The plasma viral load was assessed using the Abbott RealTime HIV (m2000sp) viral load test (Abbott Molecular, IL, USA), with a lower detection limit of 40 copies/mL. Standard flow cytometry was used to measure absolute CD4 cell counts in whole blood samples using a Beckman Coulter Navios device (Beckman, San Jose, CA, USA).

### Flow cytometry analysis

Flow cytometry was used to analyze the phenotypes of freshly isolated cells. T cell phenotype was determined using the following fluorochrome-conjugated monoclonal antibodies from BD Biosciences, San Jose, CA: anti-CD4 FITC (clone RPA-T4, RRID: AB_2562052), and anti-CD8AF700 (clone RPA-T8, RRID: AB_396953) (15). The samples were exposed to monoclonal antibodies for 15 min at room temperature in the dark, washed, and events were monitored using a Beckman Coulter Navios instrument (Beckman, San Jose, CA, USA). T helper and cytotoxic lymphocytes were gated by positive surface staining for CD4 and CD8 and were expressed as a percentage of gated lymphocytes (16).

### Definition of surgical site infection

The risk of SSI was the endpoint variable in this study. The definition and classification of SSI were based on those of the American Academy of Orthopaedic Surgeons (AAOS) (17, 18): an infection related to surgery that occurs within 30 days after surgery without an implant or within 1 year after surgery with an implant (such as a fracture internal fixation system, vertebral pedicle screw, intervertebral fusion device, artificial intervertebral disc, or prosthesis.), including superficial incision infections, deep incision infections, and organ/tissue space infections.

### The other covariates

The exposure variables were the levels of CD4 T lymphocytes and the CD4/CD8 ratio. We also collected data for other potential risk factors for SSI, which fell into three categories: demographic characteristics, surgery-related information, and preoperative laboratory examinations. Specifically, demographic characteristics included age, sex, duration of HIV infection, ART treatment, and comorbidities (such as hepatitis and diabetes); surgery-related characteristics included fracture site, injury type (closed or open), surgery duration, general or local anesthesia, and blood loss (mL); and preoperative laboratory examinations, including hemoglobin (HGB), globulin, CD4+ T lymphocyte count, CD8+ T lymphocyte count, and HIV viral load. Patient data were extracted from the HIS electronic medical record system and the data for each patient were recorded and reviewed by two or more inpatient doctors.

### Statistical methods

We analyzed the data using R version 4.1.3 software (https://www.R-project.org). Differences were considered statistically significant if the p-value was < 0.05. The data were summarized as the proportion for categorical variables. Categorical variables were compared using the chi-square test.

A logistic regression model was used to evaluate the relationship between the CD4 count, CD4/CD8 ratio, and SSI. We then conducted univariate and multivariate analyses of SSI. We constructed three logistic regression models. In Model 1 (the crude model), non-covariates were adjusted; in Model 2, age and gender were adjusted; and in Model 3, all covariates were adjusted. Furthermore, we divided the CD4 count data into three groups based on cutoff points of 200 and 350. Similarly, the ratio data were divided into three groups based on values of 0.5 and 1.0. Restricted cubic splines were used to explore non-linear relationships. We used smooth curve fitting and generalized additive models to explore the threshold saturation effect of the CD4/CD8 ratio on the risk of SSI and to determine the inflection point.

## Results

### Demographic characteristics of patients

There were 23 cases of perioperative SSI in our research population, and the majority (89.8%) of the HIV-positive patients were male. Most patients were found to be HIV-positive upon admission for injury; however, they were unaware of their HIV status and had not received timely antiviral treatment (86.57%). Many HIV-positive patients tested positive for hepatitis B or C as well, which may be because these infections are also sexually transmitted. Furthermore, the HIV-positive patients tended to have a higher incidence of lower limb fractures. Open fractures accounted for 11.1% of these cases (**Table 1**).

**Table 1:** Demographic characteristics of patients.

Variable	Overall (N=216)	No (N=193)	Yes (N=23)	P-value
Gender (%)				
Male	194 (89.81)	173 (89.64)	21 (91.30)	1.000
Female	22 (10.19)	20 (10.36)	2 (8.70)	
Age (%)				
<30 years	39 (18.06)	34 (17.62)	5 (21.74)	0.842
≥30 years	177 (81.94)	159 (82.38)	18 (78.26)	
Duration of HIV Infection (%)				
Diagnosed at fracture	92 (42.59)	81 (41.97)	11 (47.83)	0.465
<2 year	34 (15.74)	29 (15.03)	5 (21.74)	
≥2 year	90 (41.67)	83 (43.01)	7 (30.43)	
Hepatitis (%)				
No	172 (79.63)	158 (81.87)	14 (60.87)	**0.037**
Yes	44 (20.37)	35 (18.13)	9 (39.13)	
Diabetes (%)				
No	188 (87.04)	169 (87.56)	19 (82.61)	0.733
Yes	28 (12.96)	24 (12.44)	4 (17.39)	
ART (%)				
No	187 (86.57)	166 (86.01)	21 (91.30)	0.704
Yes	29 (13.43)	27 (13.99)	2 (8.70)	
Fracture Site (%)				
Arm	43 (19.91)	40 (20.73)	3 (13.04)	0.678
Leg	157 (72.69)	139 (72.02)	18 (78.26)	
Spine	16 (7.41)	14 (7.25)	2 (8.70)	
Open Fracture (%)				
No	192 (88.89)	178 (92.23)	14 (60.87)	**<0.001**
Yes	24 (11.11)	15 (7.77)	9 (39.13)	
Operation Duration (%)				
<120 min	75 (34.72)	73 (37.82)	2 (8.70)	**0.011**
≥120 min	141 (65.28)	120 (62.18)	21 (91.30)	
GA_RA (%)				
RA	137 (63.43)	124 (64.25)	13 (56.52)	0.618
GA	79 (36.57)	69 (35.75)	10 (43.48)	
Blood Loss (%)				
<400 ml	118 (54.63)	109 (56.48)	9 (39.13)	0.174
≥400 ml	98 (45.37)	84 (43.52)	14 (60.87)	
Albumin (%)				
<40 g/L	80 (37.04)	67 (34.72)	13 (56.52)	0.069
≥40 g/L	136 (62.96)	126 (65.28)	10 (43.48)	
Hb (%)				
<120 g/L	54 (25.00)	45 (23.32)	9 (39.13)	0.161
≥120 g/L	162 (75.00)	148 (76.68)	14 (60.87)	
CD4 Count (%)				
<200 cells/μL	40 (18.52)	27 (13.99)	13 (56.52)	**<0.001**
≥200 cells/μL	176 (81.48)	166 (86.01)	10 (43.48)	
CD8 Count (%)				
<400 cells/μL	32 (14.81)	29 (15.03)	3 (13.04)	1
≥400 cells/μL	184 (85.19)	164 (84.97)	20 (86.96)	
RNA Load (%)				
Not detected	117 (54.17)	113 (58.55)	4 (17.39)	**<0.001**
<2*10^4^ copies/ mL	59 (27.31)	51 (26.42)	8 (34.78)	
≥2*10^4^ copies/ mL	40 (18.52)	29 (15.03)	11 (47.83)	

The bold value means that P-value is <0.05.

In our study, we observed that the distribution of CD4 and CD8 T lymphocyte counts was right-skewed and non-normally distributed, indicating that HIV-positive patients tended to have lower CD4 and CD8 counts than healthy individuals (**Supplementary File Figure 1**).

### Univariate and multivariate logistic regression analysis of SSI

After a single factor logistic regression analysis, we found that differences in the presence of hepatitis (OR = 2.90, 95%CI = 1.13-7.17), open fractures (OR = 7.63, 95% CI = 2.79-20.6), HIV viral load (OR = 4.43, 95%CI= 1.33-17.2; OR = 10.7, 95%CI = 3.40-41.0), duration of surgery (OR = 6.39, 95%CI =1.80-40.7), albumin (OR = 0.41, 95%CI = 0.17-0.98), and CD4 count (OR = 0.13, 95%CI=0.05-0.31) were of statistical significance. Among these, the presence of hepatitis, open fractures, HIV viral load, and duration of surgery were risk factors for SSI (OR > 1), whereas albumin and CD4 counts were protective factors (OR < 1) (**Table 2**).

**Table 2:** Univariate and multivariate logistic regression analyzes of surgical site infection risk.

Variable	N	Univariate analysis			Multivariate analysis		P-value
		OR	95% CI	P-value	OR	95% CI	
**Gender**				0.799			
Male	194	—	—		—	—	
Female	22	0.82	0.13, 3.11		5.89	0.40, 91.4	0.186
**Hepatitis**				**0.028**			
No	172	—	—		—	—	
Yes	44	2.90	1.13, 7.17		6.10	1.46, 28.9	**0.016**
**Diabetes**				0.520			
No	188	—	—		—	—	
Yes	28	1.48	0.40, 4.36		0.76	0.10, 4.43	0.775
**ART**				0.459			
No	187	—	—		—	—	
Yes	29	0.59	0.09, 2.16		0.07	0.00, 0.84	0.065
**Fracture Site**				0.655			
Arm	43	—	—		—	—	
Leg	157	1.73	0.55, 7.63		0.66	0.11, 5.56	0.675
Spine	16	1.90	0.23, 12.7		0.55	0.02, 11.5	0.699
**Open Fracture**				**<0.001**			
No	192	—	—		—	—	
Yes	24	7.63	2.79, 20.6		3.22	0.74, 14.1	0.114
**GA_RA**				0.471			
RA	137	—	—		—	—	
GA	79	1.38	0.56, 3.31		0.77	0.21, 2.68	0.686
**HIV Infection Duration**				0.462			
Diagnosed at fracture	92	—	—		—	—	
<2 year	34	1.27	0.37, 3.82		3.38	0.55, 22.1	0.187
≥2 year	90	0.62	0.22, 1.66		1.76	0.39, 8.31	0.462
**RNA Load (copies/ mL)**				**<0.001**			
Not detected	117	—	—		—	—	
<20000	59	4.43	1.33, 17.2		8.68	1.42, 70.2	**0.026**
≥20000	40	10.7	3.40, 41.0		19.4	3.09, 179	**0.003**
**Age (years)**				0.634			
<30	39	—	—		—	—	
≥30	177	0.77	0.28, 2.46		0.39	0.07, 2.13	0.260
**Operation Duration**				**0.002**			
<120 min	75	—	—		—	—	
≥120 min	141	6.39	1.80, 40.7		7.84	1.35, 77.9	**0.040**
**Blood loss (ml)**				0.115			
<400	118	—	—		—	—	
≥400	98	2.02	0.84, 5.06		0.47	0.11, 1.85	0.280
**Albumin (g/L)**				**0.045**			
<40	80	—	—		—	—	
≥40	136	0.41	0.17, 0.98		0.52	0.12, 2.10	0.354
**Hb (g/L)**				0.113			
<120	54	—	—		—	—	
≥120	162	0.47	0.19, 1.20		0.54	0.12, 2.53	0.423
**CD4 (cells/μL)**				**<0.001**			
<200	40	—	—		—	—	
≥200	176	0.13	0.05, 0.31		0.05	0.01, 0.23	**<0.001**
**CD8 (cells/μL)**				0.797			
<400	32	—	—		—	—	
≥400	184	1.18	0.37, 5.23		1.53	0.17, 18.6	0.718
**CD4/CD8 Ratio**	216	0.24	0.04, 0.97	**0.045**	4.17	0.53, 33.8	0.167

The bold value means that P-value is < 0.05.

When all variables were included in the multivariate logistic regression analysis, we found that hepatitis (OR = 6.10, 95%CI = 1.46-28.9), HIV viral load (OR = 8.68, 95%CI = 1.42-70.2; OR = 19.4, 95%CI = 3.09-179), duration of surgery (OR = 7.84, 95%CI = 1.35-77.9), and CD4 count (OR = 0.05, 95%CI = 0.01-0.23) remained statistically significant (**Table 2**).

### The relationship between CD4 count and SSI

**Figure 2:**
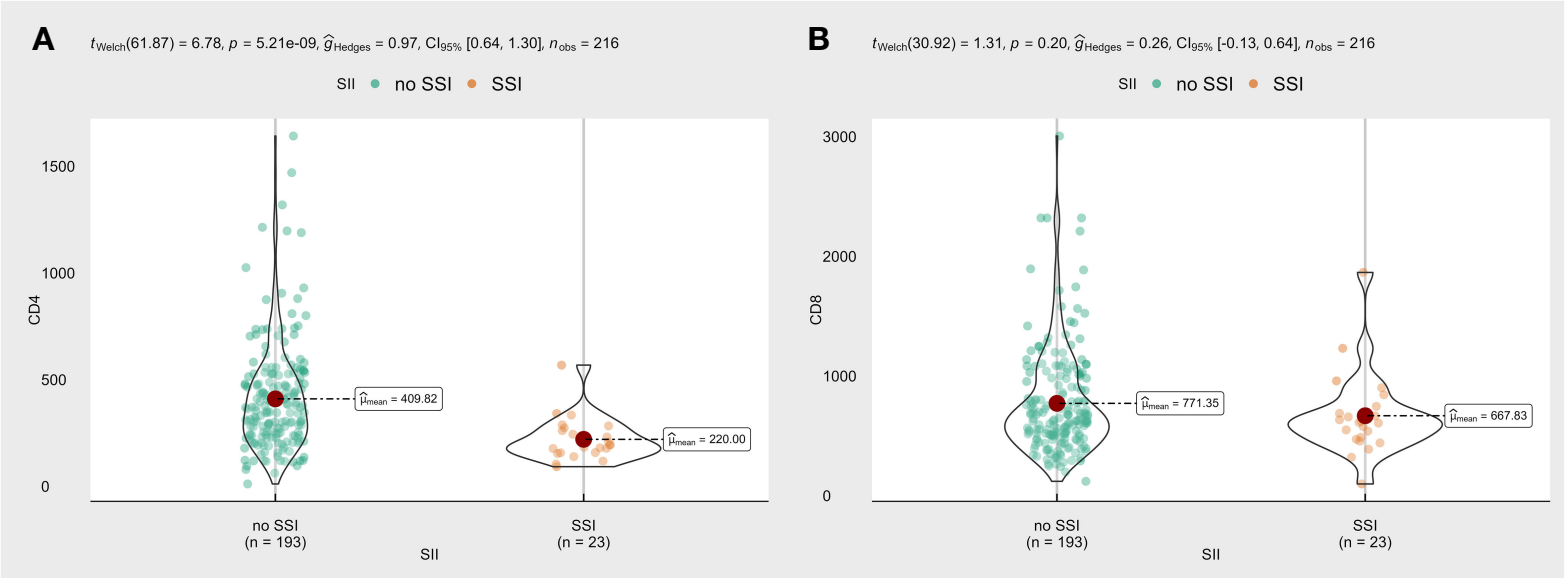
**(A)** The violin plot of CD4 cells in the SSI group or not; **(B)** The violin plot of CD8 cells in the SSI group or not.

Our results suggested that CD4 counts were lower in the SSI group than those in the non-SSI group (**Figure 2A**), and this difference was statistically significant. However, there was no difference in CD8 counts between the two groups (**Figure 2B**).

**Figure 3:**
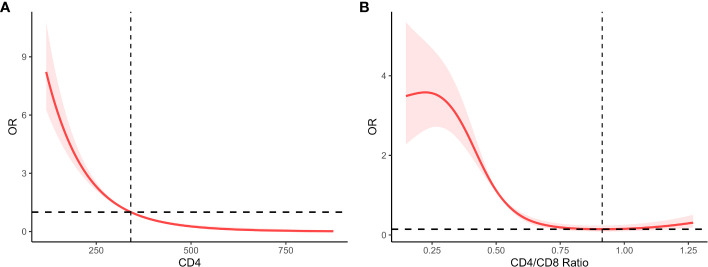
**(A)** Linear correlation plot of CD4 and SSI after adjustment; **(B)** The RCS for CD4/CD8 ratio and SSI after adjustment.

As shown in **Table 3**, our results indicated that there was a negative correlation between CD4 count and the risk of SSI after adjusting for all covariates (OR = 0.992, 95%CI = 0.985-0.997, P-value=0.011). In other words, lower CD4 T lymphocyte counts were associated with a higher risk of SSI during the perioperative period (**Figure 3A**). Further, we transformed the CD4 count from a continuous variable to a categorical variable for analysis to explore whether this relationship exists stably. Compared to patients with CD4 cell counts of less than 200, those with CD4 cell counts of greater than 350 had a 98.8% reduced risk of SSI (OR = 0.012, 95%CI = 0.001, 0.130, P-value=0.002, P for trend < 0.001).

**Table 3:** The relationship between CD4 count and the risk of surgical site infection.

	Crude Model	P-value	Model 1	P-value	Model 2	P-value
	95% CI		95% CI		95% CI	
CD4	0.993(0.988,0.996)	<0.001	0.992(0.987,0.996)	<0.001	0.992(0.985,0.997)	0.011
CD4 group						
<200	ref		ref		ref	
200-350	0.283(0.105,0.732)	0.010	0.228(0.077, 0.622)	0.005	0.168(0.035, 0.685)	0.02
>350	0.021(0.001,0.111)	<0.001	0.016(0.001, 0.090)	<0.001	0.012(0.001, 0.130)	0.002
P for Trend		<0.001		<0.001		<0.001

Crudel model: non-covariates were adjusted.

Model 1: Age, Gender were adjusted.

Model 2: Age, Gender, Hepatopathy, DM, ART, Open_fracture, GA_RA, CD8, infection_time, viral load, operation_time, bleed, Albumin, Hb were adjusted.

### The non-linear relationship between the CD4/CD8 ratio and SSI risk

As shown in **Table 4**, we found that there was a non-linear relationship between the CD4/CD8 ratio and the risk of SSI (OR = 1.183, 95%CI = 0.166-5.985, P-value=0.850), Further, we transformed the CD4/CD8 ratio from a continuous variable to a categorical variable for analysis to explore whether there is a curvilinear relationship. Compared to patients with CD4/CD8 ratio less than 0.5, those with 0.5-1.0 had the 84.7% reduced risk of SSI (OR = 0.153, 95%CI = 0.028, 0.601, P-value = 0.015, all P for trend < 0.05).

**Table 4:** The relationship between CD4/CD8 ratio and the risk of surgical site infection.

	Crude Model	P-value	Model 1	P-value	Model 2	P-value
	95% CI		95% CI		95% CI	
CD4/CD8 ratio	0.235(0.042,0.974)	0.070	0.228(0.039,0.983)	0.071	1.183(0.166, 5.985)	0.850
Ratio group						
under_0.5	Ref		ref		ref	
0.5-1.0	0.158(0.036,0.486)	0.004	0.150(0.034,0.473)	0.004	0.153(0.028, 0.601)	0.015
over_1.0	0.149(0.008,0.768)	0.069	0.133(0.007,0.727)	0.060	0.289(0.014, 2.207)	0.294
p for trend		0.003		0.003		0.030

Crudel model: non-covariates were adjusted.

Model 1: Age, Gender were adjusted.

Model 2: Age, Gender, Hepatopathy, DM, ART, Open_fracture, GA_RA, infection_time, viral load , operation_time, bleed, Albumin, Hb were adjusted.The red value means that P-value is < 0.05.

Furthermore, we used a four-point restricted cubic spline (RCS) to fit the curve. As shown in **Figure 3B**, our study shows a curvilinear relationship between them, an inverse ‘S’-shaped, ‘threshold-saturation’ effect. We also calculated the inflection point, in other words, at which the CD4/CD8 ratio was less than 0.913 and the risk of surgical site infection increased dramatically. The risk of SSI remained low when the CD4/CD8 ratio was greater than 0.913 or less than 0.221.

## Discussion

Our study found that the SSI rate in HIV-positive patients was 10.6%. However, previous studies have shown that the rate of SSI in patients with AIDS during the perioperative period can be as high as 30–75%, and the rate in HIV-negative patients is approximately 1–5% (19–21). In our study, the incidence of SSI was much lower than that reported in other domestic and foreign studies. We speculate that this may be due to our standardized preoperative preparation, including regular antiviral treatment before surgery, as well as the prophylactic use of antibiotics within 24 h before and 48 h after surgery.

Our research has shown that a decrease in CD4 T lymphocyte counts can lead to a decline in immune function, which increases the risk of SSI. Our conclusions are consistent with those of previous studies similar to our own (6, 10, 11, 20, 21). This is because CD4 T lymphocytes are a key type of cell in the immune system that help fight infections. Specifically, through antigen recognition, activation of immune responses, differentiation into specialized subsets, support of antibody production, and formation of memory cells, CD4+ T cells play a central role in orchestrating and coordinating the immune response against infections. These are some of the ways in which CD4+ T cells contribute to fighting infections. However, the CD4 T lymphocyte counts are reduced due to the viral attack, which results in a decline in immune function in HIV/AIDS patients. Therefore, a decrease in CD4 T lymphocyte count may increase the risk of SSI.

Although it is generally believed that a high CD4 count is a protective factor against SSI based on the results of previous studies, there is no consensus on the minimum CD4 count required to protect against infections. For example, Abalo et al. (6) found that CD4 counts below 500 were associated with SSI in 36 HIV-positive patients who underwent surgery for orthopedic trauma. In addition, an analysis of 64 HIV-positive patients with fractures indicated that CD4 counts below 300 were associated with postoperative infections (11). Previous Chinese studies have usually used values of 200 and 500 as cut-off points for CD4 counts. Similarly, previous studies have pointed out that the lower CD4 counts are a risk factor for SSI in patients with fractures (21–23). This conclusion was consistent with HIV-positive patients undergoing abdominal surgery (10, 24).

However, other studies have reached different conclusions, some researchers thought the CD4 count was not related to an increased risk of SSI in 77 HIV-positive women who underwent hysterectomy (25). Another study found that the relationship between HIV, CD4 count, viral load, and surgical site infection was not robust and was uncertain after sensitivity analysis in a meta-analysis of 18 cohort studies (8). Similarly, whether or not to extract teeth in HIV-positive patients was unrelated to CD4 count in 231 HIV-positive patients who underwent tooth extraction (26).

Furthermore, we explored the relationship between the CD4/CD8 ratio and the risk of SSI and found that the CD4/CD8 ratio may be a useful predictor of SSI risk. Moreover, we suggest that doctors should ensure that the CD4/CD8 ratio is at least 0.9 before the internal fixation of fractures to reduce the risk of SSI. The CD4/CD8 ratio could be a useful indicator of postoperative sepsis in HIV-infected patients undergoing abdominal surgery. A previous study found that when the CD4+ T lymphocyte count was consistently above 200 and the CD4/CD8 ratio was above 0.15, the risk of sepsis during abdominal surgery was reduced (27). Low ratios were associated with poorer survival in PLWH after analyzing 67 patients with right colon cancer (RCC) who underwent surgery (28). Moreover, this ratio not only predicts patient prognosis but can be used as a predictor of mortality in HIV-positive patients. Overall, some researchers have concluded that a lower CD4/CD8 ratio is associated with an increased risk of non-AIDS-defining events or death (29–32).

However, other researchers have different views on this matter. A previous study of 13 European and North American cohorts found little evidence that the CD4/CD8 ratio could be used to predict overall mortality after adjusting for other factors (33). In addition, AIDS-related mortality decreased as the CD4/CD8 ratio increased and the CD8 count decreased, and there was almost no evidence that the CD4/CD8 ratio or CD8 count had prognostic value for non-AIDS mortality. The Swiss Cohort Study indicated that an increase in the CD8 cell count may be a moderate risk factor for stroke, but that CD4 counts, CD8 counts, and the CD4/CD8 ratio were not associated with an increased risk of coronary artery disease (34). The CD8 count was found to be a predictor of clinical progression, but the CD4/CD8 ratio could not be used to predict an increased risk of death within seven years. Overall, many researchers do not agree on this issue (35).

We first reported that HIV-positive patients with hepatitis B or C were at higher risk of developing SSI than HIV-negative patients. This may be due to the immunocompromised state of HIV-positive individuals, which makes them more vulnerable to infections. This highlights the importance of screening for and treating hepatitis in HIV-positive patients before surgery to reduce the risk of SSI. Co-infection with the hepatitis c virus (HCV) was significantly associated with SSI in a cohort study of 305 Italian patients for the first time in 2009 (7). A comparison of four patient groups (patients with HIV, HCV, HBV, or HIV and HBV or HCV who underwent TJA) showed that the patients with HIV, HBV, or HCV had an increased risk of postoperative medical and surgical complications after TJA. However, patients with HBV or HCV infections had a higher risk of infection within 90 days than patients with HIV (36). The risk of revision was higher, but the risk of infection did not appear to increase in patients who were only infected with HIV. Up to 28% of HIV-positive patients had hepatitis C, and patients with hepatitis C had an increased risk of fracture in a retrospective study of 1981 HIV-positive patients in Massachusetts, USA (37).

This is the first and most innovative research on HIV-positive patients with fractures, and reports in this area are rare, especially in China. The Chinese scholar examined 175 clinical records and gene sequences from HCV-infected patients, including 89 (51%) HIV/HCV co-infected patients (38). They found that HCV genotypes 6a or 3a appeared most frequently in HIV/HCV co-infections and that fibrosis increased with HCV genotype 3a infections in southeast China. However, in an HIV-negative population, HIV or HCV infection was not associated with surgical site infection in a multicenter study of 1442 patients with ankle fractures (39).

The extent to which albumin is present has been shown to be a strong predictor of SSI in HIV-positive patients (23–25, 40). By providing essential nutrients, maintaining proper fluid balance, modulating inflammation, exerting antioxidant effects, and supporting immune function, albumin plays a crucial role in wound healing and immune function, and low levels of albumin can impair these processes. In addition, HIV-positive patients with low albumin levels often have other underlying conditions, such as malnutrition and inflammation, which can further increase the risk of SSI. Therefore, it is important for surgeons and healthcare providers to carefully monitor albumin levels in HIV-positive patients before and after surgery and implement interventions such as nutritional support and infection prevention measures to reduce the risk of SSI.

According to our study, HIV-positive patients with high viral loads, longer operative durations, and open fractures were more likely to develop SSI after surgery than those with low viral loads. This is consistent with the conclusions drawn from previously reported research (8, 21). These findings suggest that controlling the HIV RNA load before surgery may be an important factor in reducing the risk of SSI in HIV-positive patients. Antiretroviral therapy (ART), which is the standard treatment for HIV, can help reduce the viral load and improve immune function. Treating HIV-positive patients with open fractures requires prophylactic antibiotics and proper wound care to prevent SSI from developing in open fractures. Healthcare professionals should consider ways to optimize the efficiency of surgeries and minimize their duration, such as using minimally invasive techniques or reducing the number of surgical team members, to reduce the risk of SSI in HIV-positive patients.

There is currently no consensus among researchers on whether CD4 counts and the CD4/CD8 ratio can be used as predictors of SSI after fracture surgery in HIV-positive patients. Our study has several strengths. First, our sample size was significantly larger than those in previous studies to reduce bias and make the conclusions more reliable. Second, there are relatively few similar reports in the field of orthopedics, especially in China, and our study is the first and most innovative. Finally, we used smooth curve fitting and searched for inflection points in the study, which means that our results can provide important guidance for practical clinical work. The limitations of our study are that, compared to other large-cohort studies, the sample size was small, which may have resulted in certain errors, and the follow-up time was short. We believe that a multicenter study in China would provide the most convincing results.

## Conclusion

Our study showed that the CD4/CD8 ratio can be used as a predictor for SSI in HIV-positive patients with fractures. The risk of SSI in HIV-positive patients increased significantly during the perioperative period when the CD4/CD8 ratio was lower than 0.913. This suggests that it is necessary for doctors to reconstruct the immune function of HIV-positive patients before surgery.

## Data availability statement

The raw data supporting the conclusions of this article will be made available by the authors, without undue reservation.

## Ethics statement

This study was approved by the Ethics Committee of the Beijing Ditan Hospital, Capital Medical University. All procedures involving human participants were performed in accordance with the institutional and/or National Research Council ethical standards and the 1964 Declaration of Helsinki and its subsequent amendments, or equivalent ethical standards.

## Author contributions

BL performed data analysis and wrote the manuscript. KL, RZ, and SL contributed to data collection. KL, RZ and SL performed a literature search. QZ oversaw and managed the research. All authors contributed to the article and approved the submitted version.
