# Supplementary figures and images for: The association between the CD4/CD8 ratio and surgical site infection risk among HIV-positive adults: insights from a China hospital
Source: Front Immunol. 2023 Jul 11;14:1135725. doi: 10.3389/fimmu.2023.1135725 (PMC10366603; doi:10.3389/fimmu.2023.1135725)

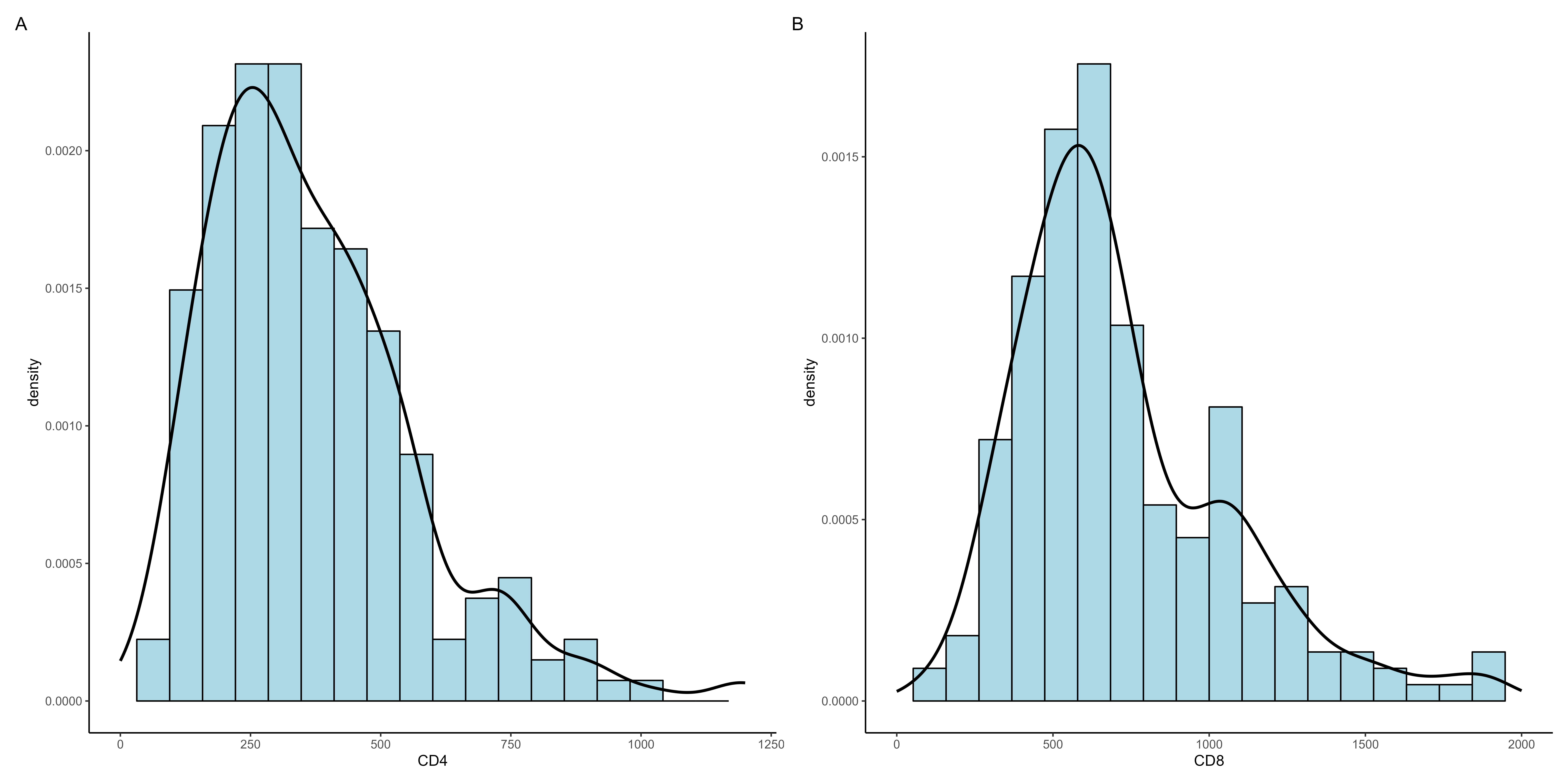

Supplement: Supplementary file 1 [file Image_1.jpeg]
